# Meeting Report: Validation of Toxicogenomics-Based Test Systems: ECVAM–ICCVAM/NICEATM Considerations for Regulatory Use

**DOI:** 10.1289/ehp.8247

**Published:** 2005-08-17

**Authors:** Raffaella Corvi, Hans-Jürgen Ahr, Silvio Albertini, David H. Blakey, Libero Clerici, Sandra Coecke, George R. Douglas, Laura Gribaldo, John P. Groten, Bernd Haase, Karen Hamernik, Thomas Hartung, Tohru Inoue, Ian Indans, Daniela Maurici, George Orphanides, Diana Rembges, Susanna-Assunta Sansone, Jason R. Snape, Eisaku Toda, Weida Tong, Joost H. van Delft, Brenda Weis, Leonard M. Schechtman

**Affiliations:** 1 European Centre for the Validation of Alternative Methods (ECVAM), Institute for Health and Consumer Protection (IHCP), Joint Research Centre of the European Commission (JRC), Ispra, Italy; 2 Bayer HealthCare AG, Wuppertal, Germany; 3 Hoffmann-La Roche, Basel, Switzerland; 4 Environmental Health Centre, Health Canada, Ottawa, Ontario, Canada; 5 Physico-Chemical Exposure, IHCP, JRC, Ispra, Italy; 6 TNO, Utrecht, the Netherlands; 7 QIAGEN, Hilden, Germany; 8 U.S. Environmental Protection Agency, Washington, DC, USA; 9 National Institute of Health Sciences, Tokyo, Japan; 10 Health Safety Executive, London, United Kingdom; 11 Syngenta, Macclesfield, United Kingdom; 12 European Molecular Biology Laboratory, European Bioinformatics Institute, Hinxton, Cambridge, United Kingdom; 13 AstraZeneca, Brixham, United Kingdom; 14 Organisation for Economic Co-operation and Development, Paris, France; 15 Food and Drug Administration, National Center for Toxicological Research, Jefferson, Arkansas, USA; 16 University of Maastricht, Maastricht, the Netherlands; 17 National Institute of Environmental Health Sciences, National Institutes of Health, Department of Health and Human Services, Research Triangle Park, North Carolina, USA; 18 U.S. Interagency Coordinating Committee on the Validation of Alternative Methods, Research Triangle Park, NC, USA; 19 U.S. Food and Drug Administration, National Center for Toxicological Research, Rockville, Maryland, USA

**Keywords:** acceptance, alternatives, biomarker, predictive test, regulatory use, standardization, toxicogenomics, toxicology, validation

## Abstract

This is the report of the first workshop “Validation of Toxicogenomics-Based Test Systems” held 11–12 December 2003 in Ispra, Italy. The workshop was hosted by the European Centre for the Validation of Alternative Methods (ECVAM) and organized jointly by ECVAM, the U.S. Interagency Coordinating Committee on the Validation of Alternative Methods (ICCVAM), and the National Toxicology Program (NTP) Interagency Center for the Evaluation of Alternative Toxicological Methods (NICEATM). The primary aim of the workshop was for participants to discuss and define principles applicable to the validation of toxicogenomics platforms as well as validation of specific toxicologic test methods that incorporate toxicogenomics technologies. The workshop was viewed as an opportunity for initiating a dialogue between technologic experts, regulators, and the principal validation bodies and for identifying those factors to which the validation process would be applicable. It was felt that to do so now, as the technology is evolving and associated challenges are identified, would be a basis for the future validation of the technology when it reaches the appropriate stage. Because of the complexity of the issue, different aspects of the validation of toxicogenomics-based test methods were covered. The three focus areas include *a*) biologic validation of toxicogenomics-based test methods for regulatory decision making, *b*) technical and bioinformatics aspects related to validation, and *c*) validation issues as they relate to regulatory acceptance and use of toxicogenomics-based test methods. In this report we summarize the discussions and describe in detail the recommendations for future direction and priorities.

Toxicogenomics, an emerging field in molecular toxicology, offers the promise of new approaches to identify and characterize such factors as the biologic activity of new and existing chemicals and drugs and could play an important role in hazard assessment for human health. This revolutionary field can potentially affect many scientific and medical areas, including the development of a new generation of alternative predictive testing and screening methods that could lend themselves to the reduction, refinement, and replacement of animals used for such purposes.

The European Centre for the Validation of Alternative Methods (ECVAM), the U.S. Interagency Coordinating Committee on the Validation of Alternative Methods (ICCVAM), and the National Toxicology Program Interagency Center for the Evaluation of Alternative Toxicological Methods (NICEATM) are currently investigating the specific considerations necessary for adequate validation of toxicogenomics-based test methods. The primary objective of ECVAM and ICCVAM/NICEATM is to facilitate development, validation, and regulatory acceptance of new, revised, and alternative test methods that reduce, refine, and replace the use of animals (referred to as the three Rs; Russell and Burch 1959) in testing while maintaining and promoting scientific quality and the protection of human health, animal health, and the environment. The efforts of such organizations as ICCVAM/NICEATM and ECVAM have helped foster the principles of the three R’s and have contributed to progress in the use of alternative methods for regulatory, research, and educational purposes.

Experience in the validation of conventional alternative test methods has led to an understanding that new and innovative approaches likely will be necessary to standardize test methods based on toxicogenomics and to evaluate the scientific validity and regulatory applicability of such test methods. It is envisioned that the entire validation process will be more complex and challenging than that typically encountered thus far for other alternative test methods. This is because not only will the technology itself need to be standardized and validated, but the methods that are based upon the technology and their predictive aspects will also need to undergo validation if they are to be employed in regulatory decision-making processes. In addition the validation process must be able to accommodate the anticipated rapid changes in technology that could affect the performance of the test method and its reliability for a specific purpose.

Toxicogenomics-based methods are being widely applied in toxicology and biomedical research. Because data are already being generated using these technologies, it is both timely and important to address the subject of validation now with the aim of establishing a foundation that will facilitate future regulatory acceptance of scientifically validated toxicogenomics-based test methods. By addressing the critical validation issues early, and in parallel with the evolutionary and maturation phases of the technologic development of toxicogenomics-based methods, it should be possible to preempt many potential pitfalls and data gaps encountered with retrospective method evaluations that could impede validation of this promising research and regulatory tool. Such a strategy will also facilitate early buy-in and confidence in the technologies by the regulatory arena in its quest for new, improved, and relevant methods by which to help ensure human health, protect the environment, and demonstrate responsiveness to animal welfare issues.

In consideration of all these related issues, ECVAM and ICCVAM/NICEATM held the first of a planned series of workshops to address the validation principles that lend themselves to toxicogenomics-based test methods, for example, gene expression technologies and associated bioinformatics. Given the complexity of the rapidly evolving toxicogenomics field, a variety of issues were addressed. These included but were not limited to *a*) differences in and evolution of technology platforms including changes in genome coverage for model species; *b*) quality assurance (QA) and Good Laboratory Practice (GLP) compliance; *c*) technology standardization, transferability, and reproducibility; *d*) relevance to *in vivo* biological responses; *e*) yardsticks against which toxicogenomics responses should be measured; *f* ) data evaluation, statistical approaches, and databases; *g*) validation approaches; and *h*) regulatory acceptability.

To begin to examine these complex issues, three breakout groups were formed. Each group concentrated on different aspects of the validation of toxicogenomics-based test methods, and the discussions were shared with the other participants in plenary sessions. The three focus areas were *a*) biological validation of toxicogenomics-based test methods for regulatory decision making, *b*) technical and bioinformatics aspects related to validation, and *c*) validation issues as they relate to regulatory acceptance and use of toxicogenomics-based test methods.

## Validation of Toxicogenomics: Focus on the Biological Systems

The biological issues related to the validation of toxicogenomics-based test methods involved two strategies proposed for developing and validating such methods so that they can be employed to support regulatory decision making. One strategy involves phenotypic anchoring of gene expression changes to identify molecular mechanisms and candidate bio-markers of toxicity (i.e., single genes, proteins, or biological pathways). A second strategy involves the identification and validation of predictive gene expression signatures of toxicity. Validation considerations specific to data quality and cross-platform and interlaboratory variability that are common to both strategies were identified. It is acknowledged that any new toxicogenomics-based methods will need to address established validation criteria for determination of reliability and relevance (Balls et al. 1995; ICCVAM 1997, 2003) as well as articulate the advantages and limitations of a given toxicogenomics-based test method. In addition biological validation of such a test method, that is, assessment of the concordance of gene changes with biological events, is essential but is contingent upon validation of the technology itself, which is addressed elsewhere in this article.

### Strategy 1: use of toxicogenomics data to define mechanism and identify biomarkers.

Toxicogenomics offers the opportunity to enhance existing toxicity prediction strategies through elucidation of biological mechanisms around critical events. This sentiment is captured in the recent U.S. Environment Protection Agency (EPA) and U.S. Food and Drug Administration (FDA) strategies regarding the inclusion of genomics data in submissions of regulated substances (U.S. EPA 2002; U.S. FDA 2005). Although these agencies currently preclude basing regulatory decision making on genomics data alone, they do encourage the voluntary submission of well-documented, quality genomics data. Both agencies are considering the use of submitted data on a case-by-case basis for assessment purposes (e.g., to help elucidate mechanism of action or contribute to a weight-of-evidence approach) or for populating relevant comparative databases by encouraging parallel submissions of genomics data and traditional toxicologic test results. This approach is appropriate given the state of scientific knowledge of toxicogenomics and the requisite need for a clear understanding of the toxicologic relevance of the gene expression signals detected by this technology. There is a small but rapidly increasing number of published reports demonstrating a linkage between gene expression changes and adverse phenotypic changes (Huang et al. 2003; Orphanides 2003). These reports provide qualitative evidence of the power of genomics to link phenotype with gene expression, thereby contributing to an understanding of mechanism of action. Some such reports demonstrate the predictive power of these data to classify compounds. However, they fail to address adequately quantitative dose- and time-dependent (e.g., threshold) responses that are the hallmark of toxicologic evaluation, making their immediate acceptance in regulatory arenas circumspect.

Nonetheless, toxicogenomics data may eventually be useful in hazard and risk assessment if data quality and validity can be adequately substantiated. Some regulators are finding that these data have the potential to add to the body of knowledge about compound mechanism of action. With appropriate dose- and time-dependent measurements, gene and protein changes can be used to mark the molecular events that occur as an organism moves through the continuum from exposure to response. The obvious benefit is the identification of early markers of response, including responses that mark the point of departure from adaptation to toxicity. In addition, it may be possible to detect unforeseen effects at very low doses or in unexpected tissues (Brown et al. 2002). This is important because changes in gene or protein expression alone are not sufficient to differentiate toxicity from biologic adaptation after exposure to an exogenous compound. The challenge for predictive toxicology is to link changes in gene and protein expression to sequential changes in phenotype, both adaptive and adverse, in a manner that is consistent with the underlying biologic mechanisms. For example, gene expression profiling has been used to classify hepatotoxins based on mechanism of action and to differentiate early, presumably adaptive, responses from later responses that are reflective of toxicity (Hamadeh et al. 2002a, 2002b; Waring et al. 2001, 2003). The gene expression changes correlated well with changes in histopathology and clinical chemistry, supporting the liver as target organ for the test compounds.

Although good technical progress has been made in recent years, additional proof-of-principle studies are needed for the regulatory community to become more accepting of the use of toxicogenomics data as part of the regulatory decision-making process. It would be important to demonstrate, for instance, that toxicogenomics not only can confirm what is already known about specific compounds and toxic end points (i.e., phenotypic anchoring) but also can accurately predict toxicity for unknown compounds. The task is to present regulatory scientists with new knowledge gained from toxicogenomics approaches in a familiar context. Ideally, at least in the short term, the focus will be the identification of single, or small sets of, genes or proteins that serve as biomarkers of response, as opposed to signatures of response that are the typical output of microarray experiments. Simple biomarkers of response are favored over complex expression signatures because they are familiar in toxicology assessment, are easy to maintain over time (e.g., are independent of the microarray platform), and can be readily validated. Validation strategies for toxicogenomics-based markers can be modeled after protocols for existing biomarkers. Thus, global gene expression technologies such as microarrays can be used to identify a specific gene marker, or a suite of markers, that can then be validated by conventional methods such as Northern blot analysis, *in situ* hybridization, and quantitative polymerase chain reaction. This approach has advantages because regulatory agencies such as the U.S. FDA have proposed procedures to address gene and protein biomarkers, and other organizations, such as the Organisation for Economic Co-operation and Development (OECD 2005), are embarking on establishing similar guidance (Supplemental Material, Section 1; http://ehp.niehs.nih.gov/members/2005/8247/suppl.pdf).

Proof-of-principle studies could be conducted concurrently with existing regulatory test methods using similar samples of test compounds. In such situations, it may be appropriate to use *in vivo* systems, which are widely accepted by the regulatory community. Parallel *in vitro* studies could be conducted in situations where an appropriate test system is available. It may be wise to focus initial efforts on defining relationships between gene expression changes and toxicity for individual compounds or compound classes with well-defined end points. The experimental design should address conventional aspects of dose and time (dose response), species and strain susceptibility, group size and sex, and selection of end points for study (e.g., histopathology, clinical chemistry). Numerous commercial microarray platforms offer genomewide coverage for model systems such as rat, mouse, *Caenorhabditis elegans*, and humans. Commercial microarrays are also available for genes that are highly expressed in specific tissues (e.g., liver, breast) and during specific biological processes such as metabolism (e.g., P450 enzymes). Both genomewide and dedicated arrays can be used with RNA samples from *in vivo* and *in vitro* (tissue and cell culture) systems, enabling parallel studies to be conducted with a single microarray platform. This is important because the results of microarray experiments can vary depending on the array design and the selection and performance of gene probes on the array. Encouraging results on cross-platform comparisons and between-laboratory reproducibility are now emerging (Bammler et al. 2005; Chu et al. 2004; Irizarry et al. 2005; Larkin et al. 2005; Yauk et al. 2004). Toxicogenomics studies conducted in parallel and comparative systems can demonstrate the biologic relevance of *in vitro* models as surrogates for *in vivo* models without the need to address cross-platform (technologic) issues (Boess et al. 2003; Huang et al. 2003). Although initial efforts should focus on defining simple gene and protein bio-markers for specific compound classes, end points, and model systems, the end goal is to establish a compendium of compound-specific knowledge that transcends technology platform. Ideally, the markers should be robust enough to withstand technologic advances in toxicology that add to the existing knowledge about the compound. Once sufficient and adequately validated data are available, toxicogenomics can become part of a hierarchical approach to compound assessment.

The use of toxicogenomics to identify (screen) compounds with the potential to cause adverse effects may present opportunities to reduce the need for full animal tests, or perhaps refine animal use, and/or reduce the numbers of animals needed when *in vivo* tests are necessary. Of course, the statistical power of any test will influence the number of animals used in an *in vivo* test as well. Screening-type assessments may be appropriate for priority setting, dose setting, chemical ranking, and so forth. The extent of validation required for screening tests may be different than that required for full replacement tests because negative compounds might still undergo full animal testing. Establishing a compendium of compound-specific information will enable regulators and sponsors to access what is known about a compound across multiple test systems, species, and end points, thereby improving the biological relevance of regulatory decisions to safeguard human health and the environment.

### Strategy 2: use of gene expression signatures to predict toxicity.

Toxicogenomics holds great promise for improving predictive toxicologic assessments. Gene expression profiling has been used to classify compounds by chemical class and mechanism (Hughes et al. 2000; Scherf et al. 2000; Steiner et al 2004; Thomas et al. 2001), tumors by origin and type (Chung et al. 2002), and breast cancer patients for follow-up chemotherapy (van ‘t Veer et al. 2002). In all cases, classification was based on a set of discriminatory gene elements, between 10 and several hundred, identified from a larger pool of genes on a microarray. The pattern of gene expression, not the measurement of a single or a small set of genes, was the basis for classification. A variety of gene expression analysis algorithms were used to discriminate samples based on gene expression signature. In all cases, the compound class or tumor status was known *a priori*, and gene expression signatures for known samples were used to predict classification for other known but blinded samples (Blower et al. 2002; Brindle et al. 2002). Such models are currently being developed in the private sector (e.g., Gene Logic, Iconix) and are commercially available but cannot, as yet, be exploited by regulators and the scientific community because the underlying data sets and algorithms have not been made available outside the private sector.

Predictive model development will require an extensive “training” set of gene expression measurements for classes of model compounds in a variety of test systems, both *in vivo* and *in vitro*, at multiple doses and time points. Initial studies can be conducted concurrently with conventional testing systems as a way to confirm model predictions. In the short term, it is unlikely that sufficient data will be available for gene expression signatures to replace conventional approaches. Until then, such data can be used as part of a hierarchical approach to toxicity testing in conjunction with accepted methods routinely used for regulatory purposes. In the long-term, sufficient data should accumulate from well-designed validation studies such that gene expression signatures could be part of a battery of tests that reduce or replace animal procedures.

Model validation will necessitate multiple independent data sets and application of sophisticated statistical approaches. Acceptance of these models will require that research and regulatory communities have access to the data analysis tools used to build the models, and that they become familiar with the limitations and uncertainties of using these complex computational models. Confidence in and acceptance of these models will also require rigorous performance standards and appropriate controls to ensure reproducibility and stability over time (see below) and adequate sensitivity and specificity to discriminate toxic from non-toxic responses. Initial model development could easily be accelerated through coordinated sector-spanning efforts. Coordinated efforts across academia, government, and industry partnerships will accelerate progress in defining gene sets that are robust and discriminatory both within and across technology platforms. This is an ideal scenario given the rapidly advancing pace of technology development.

An important aspect of any toxicogenomics validation strategy is the need to measure the range of biological variability of gene responses for a given test system. Ideally, this should be accomplished by one species, tissue, and end point at a time, in order to adequately assess cross-species differences that often hamper risk assessments. Measurements of biologic variability under baseline and toxicant-challenged conditions will enable regulators to better discriminate biologically relevant responses from baseline homeostatic fluctuation. This is an important issue for toxicogenomics, as studies conducted on cell culture populations demonstrate a wide range of biological variability in gene expression measurements for individual cells under both baseline and challenged conditions (Kuang et al. 2004). Therefore, it is necessary to define criteria to adequately address biological variability in a data submission and to establish whether the burden of maintaining these data is that of the regulator or sponsor.

The recommendations related to the biological validation of toxicogenomics-based test methods are listed in Table 1.

## Standardization and Validation of Toxicogenomics-Based Methods: Focus on the Technology

Considerations given to validation of the technology encompassed the technical and bioinformatics issues related to the validation of toxicogenomics-based test methods. The starting premise adopted was that with the availability of bioinformatics expertise, biological data generated from toxicogenomics studies could be interpreted with a high degree of confidence. The ultimate aim was to identify a strategic approach that would enable credible biological observations and consequential judicious regulatory decisions, and that this approach would be independent of the toxicogenomic platform used. Moreover, standardization and validation of toxicogenomic platforms were seen as essential for identifying and reducing technologic artifacts. Standardization would also be required to increase the certainty by which biological observations could be extrapolated across and between different microarray platforms. It is therefore important to build on the learning of previous and ongoing efforts in standardization of toxicogenomics (reviewed by Sansone et al. 2004).

**Figure 1:**
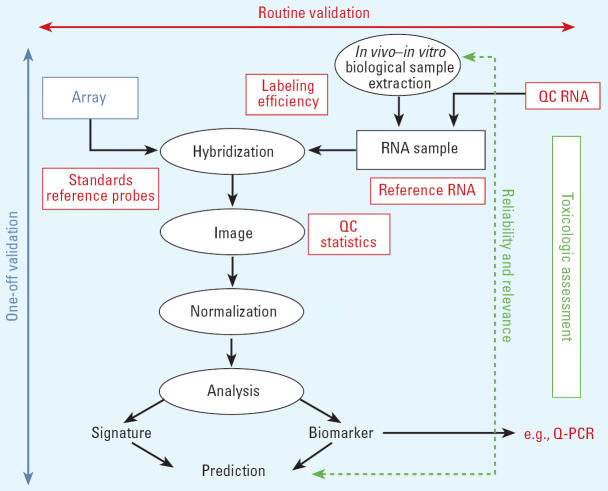
Scheme of the different steps in a toxicogenomics-based test. Three distinct levels were identified where validation is necessary: one-off validation (left), which should be performed once and is mainly related with the quality of the microarray and the instrumentation (blue); routine validation and QC (top), representing the ongoing requirements that are the responsibilities of the experimental toxicologist and the manufacturer (red); and the extent of validation necessary whenever a technical or methodologic change is introduced in the test (right): a method should meet the preestablished performance standards in order to be considered reliable and relevant as the original test method (green). Q-PCR, quantitative PCR.

Three distinct levels where validation is necessary were identified (see Figure 1 and discussion below). The first level of validation is the responsibility of the array manufacturer or provider and has to be performed only once. This can be seen as a “one-off validation” and relates to both the microarray quality and the instrumentation. The second level of validation is the responsibility of both the experimental toxicologist and the array manufacturer or provider. This can be seen as “routine validation” or best practice to allow data comparability. It encompasses quality control (QC) aspects of the critical experimental components and is a process that occurs on a regularly scheduled basis. The third level of validation, that is, determination of reliability and relevance, is needed every time a change is introduced into the test procedure. Performance standards developed based upon the original test method would serve as the criteria against which the revised method would be compared. Despite these multilevel validation needs, it was repeatedly emphasized that significant technologic development and progress in microarray platforms are still under way and that efforts to validate and standardize these technologic platforms must not be at the expense of innovation.

### One-Off Validation

The one-off validation is the responsibility of the array manufacturer or array provider. This is required to ensure that the array platform being used is robust and that the inherent variability within the platform is transparent to the user and the regulator (Figure 1). The following were identified as being necessary for microarray-based toxicogenomics to be used in regulatory assessments:

- Microarrays should be fabricated in accordance with the principles of Good Manufacturing Practice (GMP).
- Specifications and performance criteria for all instrumentation and method components should be available.
- All quality assurance/quality control (QA/QC) procedures should be transparent, consistent, comparable, and reported.
- The array should have undergone sequence verification, and the sequences should be publicly available.
- All data should be exportable in a MAGE (MicroArray and Gene Expression)-compatible format.

### Routine Validation

Routine validation is an ongoing process that is the responsibility of the experimental toxicologist and the array manufacturer or provider (Rockett and Hellmann 2004). Again, for microarray-based toxicogenomic assays to be used in regulatory decision making the following important factors were identified (Figure 1):

- Oligos, cDNAs, or clones that are arrayed should be randomly sequence-verified to ensure that no errors are introduced between batch syntheses. This verification process should be recorded and reported by the manufacturer
- All reagent components should be identified. Reagents should be prepared according to GMP and/or GLP as appropriate. Data regarding batch variability should also be recorded and reported
- Common reference RNA standards (housekeeping genes) should be adopted to facilitate comparison between array platforms. This may be achieved in collaboration with the international Microarray Gene Expression Data (MGED) Society and other related efforts (see below).

#### Biological standards.

Performance standards, test component standards, and QC measures are key components of any validation strategy for a toxicologic test method. Establishing standards is particularly important for gene expression technologies due to the inherent technologic and biological “noise” in these systems. Commonly used biological standards are reference RNAs that are competitively hybridized with the sample of interest in two-channel array formats, and *in vitro* RNA transcripts that are “spiked into” RNA samples of interest in either one-channel or two-channel array formats. Establishing accepted RNA standards will address concerns of regulatory reviewers about data quality and variability within and between laboratories and across different technology platforms. The standards will also provide a common benchmark for regulators to assess platform performance over time. To achieve this goal, we must establish standards that maintain a defined level of accuracy, sensitivity, specificity, and reproducibility across platforms.

Reference RNAs can be derived from tissue extracts, cell lines, or both and serve a variety of purposes. Workshops sponsored by governments and industry have focused on defining the specifications for reference RNAs for clinical and regulatory applications (Joseph 2004). The consensus is a that multiple RNA standards are needed to measure the accuracy, dynamic range, sensitivity, and specificity of varied technology platforms under varied conditions. Important questions are whether regulatory agencies will define preferred sources of RNA standards, and, if so, who will generate and maintain baseline information about these standards. Although the selection of a given RNA standard depends primarily on the purpose and application, all RNA standards should be tested for a clearly defined number of copies of a given sequence within an RNA preparation over some linear range (Cronin et al. 2004).

Some initiatives are raising awareness of the effects of variables that might hamper data comparability and are working toward developing best practice guidelines for microarray-based measurements (Hopkins et al. 2004). For example, recommendations for best practice in array normalization, together with performance characteristics in terms of sensitivity, accuracy, and comparability of different array platforms (cDNA and oligo, spotted and *in situ* synthesis), are beginning to emerge together with proposals for transparency and availability through publicly accessible databases (http://www.vam.org.uk). Other initiatives are considering the use of quality metrics for standardizing and validating array-based toxicogenomics measurements. The extent to which such efforts will be pursued and the impact they will have upon the standardization issues that are a necessary prerequisite to the validation exercises remain to be seen.

#### Quality assurance and Good Laboratory Practice.

GLP is intended to promote proper documentation, quality, and authenticity of toxicity test data and is required for data acceptance by regulatory agencies (e.g., U.S. FDA, U.S. EPA). At the international level, GLP has been promulgated under the OECD guidelines program (OECD 1998). As part of the progression toward regulatory acceptance, toxicogenomics experiments should ideally be conducted in accordance with GLP. However, at present, most large-scale toxicogenomics efforts are not arising from GLP-compliant laboratories, and requiring compliance for data submission could greatly hamper the technical advancement of new technologies and retard their migration into the regulatory arena. To avoid discouraging technologic progress while maintaining a level of GLP conformity, it could be argued that for research and technical development and improvement purposes, it might be acceptable if array-based studies could at least measure up to the reporting standards required by GLP. However, with the adoption of the toxicogenomics-based technologies into regulatory decision-making practices, GLP compliance undoubtedly will be expected. Procedural aspects of GLP compliance not currently captured in MIAME-Tox (minimum information about a microarray experiment for toxicogenomics) will need to be identified but can be incorporated over time. Until then, it may be possible to allow for proof-of-principle and prevalidation studies to be conducted in accordance with the “intent” of GLP practices by requiring submitters to adequately document procedures and control measures and make experimental data open to regulatory review. “Best practices” for toxicogenomics can be established until formal procedures are adopted. This may be a more realistic solution that permits the advancement of science while addressing the need for QA and QC.

### Validation as a Result of Procedural Changes

This third level of validation is necessary whenever a technical or methodologic change is introduced into the test. Such changes might, on one hand, be restricted to the microarray technology (e.g., modification or addition of sequences to a microarray, changes in data analysis procedures). Alternatively, they could involve the experimental design (e.g., dose, time, cell culture procedures). One consideration is that a distinction between minor and major procedural changes that might be incorporated into a test would help determine the extent of such validation necessary. Additionally, to facilitate the process, performance standards should be defined based upon the original validated test procedure. Minor changes would entail a demonstration of equivalence of results obtained with the modified test to that obtained from the validated test. Major changes would involve the need to define a new set of reference materials to be tested and a more extensive validation. Guidance on the use of performance standards and the elements comprising them have been published (ICCVAM 2003) and have been employed for *in vitro* dermal corrosion assessment methods (ICCVAM 2004). Such guidance can also help facilitate the establishment performance standards for toxicogenomics-based test methods in which procedural modifications have been introduced after an initial validation exercise, thereby providing a basis for the comparison of reliability and accuracy of the modified method relative to the validated and accepted reference test method.

The concept of performance standards was originally developed to evaluate the acceptability (accuracy and reliability) of proposed test methods that are based on similar scientific principles and that measure or predict the same biologic or toxic effect as an accepted (previously validated) test method. Because some regulatory authorities and international test guidelines programs (e.g., OECD) have restrictions regarding the use of proprietary test methods (methods that are copyrighted, trademarked, or patented), performance standards also allow for the development and validation of comparable nonproprietary methods based on performance standards derived from the corresponding proprietary antecedent method. Under these circumstances, performance standards allow the characteristics and functional attributes of a proprietary method or technique to be described and offer a procedure for evaluating the performance of methods claimed to be substantially similar. A method that meets the established performance standards is considered sufficiently accurate and reliable for the specific testing purpose for which it is designed and is viewed as comparable with the original test method upon which it is based. If the correct performance standards have been developed, a method for which the results have the same accuracy and reliability as the original should by definition also be as relevant as the original method.

The conceptual framework and scope of performance standards could be expanded or adapted to include innovations or advancements in areas such as microarray or protein or metabolite separation and identification technology, where proposed improvements might or might not be generally or completely analogous to those in existing systems but would still enable similar applications. Performance standards could still provide a gauge for evaluating newer or revised technologies to ensure that their reliability and accuracy were at least comparable with that of existing acceptable techniques using similar chemicals even if essential test method components (i.e., structural, functional, and procedural elements of a validated test method to which a proposed, mechanistically and functionally similar test method should adhere) were not substantially similar.

This level of validation, which does not imply that a test needs to be completely revalidated, is of extreme importance for tests based on rapidly evolving technologies. It would be a mistake to immobilize these technologies by enforcement of a strict and inflexible validation approach that would hamper progress and test improvement. Finally, a periodic reassessment of a test method’s performance (accuracy and reliability) employing established performance standards would help ensure adherence to essential test method components and the reliability and accuracy of the modified test method relative to the validated antecedent method (Hartung et al. 2004). Such assurance could be best established and reported by international validation bodies such as ECVAM and ICCVAM/NICEATM, which could track the history, performance, and validation status of a given test.

### Data Management

The lack of robust QC procedures and capture of adequate metadata has caused problems with the analysis and reproducibility of array-based transcriptomics investigations. Consequently, the international MGED Society proposed standards for publication (Nature 2002) that were designed to clarify the MIAME guidelines (Brazma et al. 2001). As a result, a number of journals now require that articles containing microarray experiments must be compliant with the MIAME standard; some also require that the data integral to the article’s conclusions be submitted to the ArrayExpress database at the EBI (European Bioinformatics Institute) (Brazma et al. 2003), GEO (Gene Expression Omnibus) at NCBI (National Center for Biotechnology Information) (Edgar et al. 2002), and CIBEX (Center for Information Biology Gene Expression database) at DDBJ (DNA Databank of Japan) (Ikeo et al. 2003)—the European, American, and Japanese database counterparts, respectively.

There is a critical need for public toxicogenomics databases because of the significant volume of data associated with these experiments, the complexity of comparing different gene annotations and splice variants across platforms, and the need for a resource for complex informatics analyses of the traditional toxicology and microarray data in parallel. However, to fully achieve the potential of this emerging interdisciplinary field, it is necessary that we move toward the establishment of a common public infrastructure for exchanging toxicogenomics data (Mattes et al. 2004). The infrastructure should address *a*) the technical problems involved in data upload, *b*) the demand for standardizing data models and exchange formats, *c*) the requirement for identifying minimal descriptors to represent the experiment, *d*) the necessity of defining parameters that assess and record data quality, and *e*) the challenge of creating standardized nomenclature and ontologies to describe biological data. The goal is also to create an internationally compatible informatics platform integrating toxicology/pathology data with transcriptomics, providing the scientific community with easy access to integrated data in a structured standard format, facilitating data analysis and data comparison, and enhancing the impact of the individual data sets and the comprehension of the molecular basis of actions of drugs or toxicants. Ultimately, such a knowledge-base could be maintained (respecting confidentiality as appropriate) as a reference for regulatory organizations to evaluate toxicogenomics and pharmacogenomics data submitted by registrants to those organizations.

The potential exists for the international development of this public infrastructure. As part of the collaborative undertaking with the International Life Sciences Institute Health and Environmental Sciences Institute (ILSI–HESI) Technical Committee on the Application of Genomics to Mechanism Based Risk Assessment (http://www.hesiglobal.org/committees), the European Molecular Biology Laboratory of the European Bioinformatics Institute (EMBL–EBI; Brazma et al. 2003; http://www.ebi.ac.uk/microarray/Projects/tox-nutri/index.html), the National Institutes of Health/National Institutes of Health National Institute of Environmental Health Sciences National Center for Toxicogenomics (NCT; Waters et al. 2003; http://www.niehs.nih.gov/nct/), and the U.S. FDA NCT (Tong et al. 2003; http://www.fda.gov/nctr/science/centers/toxicoinformatics/index.htm) have worked closely together. The respective databases are based on the international standards developed by the MGED Society (Brazma et al. 2001; Spellman et al. 2002). After the very favorable response that the MIAME received from the microarray community and key scientific journals (Ball et al. 2002, 2004; Nature 2002), the MIAME checklist was extended to describe array-based toxicogenomics experiments. The MIAME-Tox checklist (MGED 2004) is an attempt to define the minimum information required to interpret unambiguously and potentially reproduce and verify array-based toxicogenomics experiments. MIAME-Tox also supports a number of other objectives, for example, linking data from different experimental domains within a study and linking several studies from one institution and exchanging toxicogenomics data sets among public databases. The major objective of MIAME-Tox is to guide development of toxicogenomics databases and data management software. Without a sufficient depth of data in these resources, the scientific community’s opportunity to develop consensus on analysis and application of these data for risk assessment or screening may be limited. The availability of this level of information regarding platform specification, appropriate common reference standards, and the toxicologic study alone will facilitate the predictive value of toxicogenomics across different array-based platforms. This, in turn, will result in a greater appreciation of and confidence in the value of toxicogenomics within a regulatory context, such that testing strategies can be optimized, predictive alternative models can be identified, and animal use can be reduced (Supplemental Material, Section 2; http://ehp.niehs.nih.gov/members/2005/8247/suppl.pdf).

Moreover, the long-term provision of a MIAME-Tox–compliant database with a MAGE-ML (Microarray Gene Expression Markup Language) export is required for the long-term storage of toxicogenomics data. This would directly support the role of ECVAM, ICCVAM/NICEATM, and other validation bodies in the validation of toxicogenomics-based test methods.

The recommendations related to the technical and bioinformatics aspects of validation are listed in Table 2.

## Regulatory Acceptance of Validated Toxicogenomics-Based Methods

Regulatory scientists are increasingly being called upon to consider incorporation of toxicogenomics data in regulatory assessment processes that involve evaluation of potential human health or environmental hazard and risk. Those scientists will need to be able to judge the level of confidence to place in both *in vivo* and *in vitro* toxicogenomics-based test methods and the resulting data that might be submitted in support of regulatory decision making. Whether a method has been determined to be valid for a specific purpose will be an important factor for the consideration of its use for regulatory purposes. Furthermore, the level of confidence held by regulators will influence regulatory acceptance of methods and data, and will affect both the further pursuit of toxicogenomics technologies and technologic improvements and the extent of industry application of these technologies.

### Potential uses of toxicogenomics data in the regulatory area.

The potential of toxicogenomics-based methods in contributing to regulatory assessment processes is broad. Examples might include, but would not be limited to, obtaining microarray data from individual *in vivo* bioassays or *in vitro* cell or tissue-based assays or from batteries of assays, using conventional or high-throughput approaches. In accordance with the current developing state of the science, realistic possibilities for initial uses of toxicogenomics data in regulatory settings might be first in the realm of hazard assessment, such as to support chemical mechanism of action arguments. Other early uses might include aiding individual chemical/chemical mixture screening or ranking exercises to set priorities for toxicity testing or to sort chemicals into batches. These types of applications might involve identification of individual genes or gene patterns associated with particular toxic effects or pathways, adaptive responses, or metabolic pathways. However, global pattern recognition–type techniques are, as yet, not considered to be ready to fully replace traditional bioanalytical methods for predicting toxicity or elucidating information on mechanism of action or biochemical pathway component identification.

Using only human or animal *in vitro* or *in vivo* data derived from toxicogenomics technology to estimate such parameters as adverse/no adverse effect levels or to determine dose–response relationships for conducting risk assessments is regarded as a much longer term goal. However, for hazard assessment purposes, the possibility of considering toxicogenomics data along with other types of toxicologic information and data [e.g., from *in vivo* and *in vitro* studies, determinations of quantitative structure–activity relationships (QSAR) or SAR] in a weight-of-evidence approach on a case-by-case basis was not discounted. Regulatory bodies have begun to craft preliminary proposals, policies, and guidance for the submission and use of omics-type data in regulatory deliberations and to provide encouragement for the use and further development of the technology (U.S. EPA 2002; U.S. FDA 2005). Additionally, organizations such as the OECD are actively working with member countries on approaches that seek to harmonize the use of omics-derived information for hazard assessment related to health and environmental effects.

Harmonization of toxicogenomics-based test methods will first necessitate the standardization and validation of the specific test protocol(s) developed for a specific purpose(s), as conducted by international validation bodies such as ECVAM and ICCVAM/NICEATM. It will then be important for such organizations to interface with the OECD to ensure the appropriate crafting of harmonized OECD toxicogenomics-based test guidelines that are based upon standardized, adequately validated procedures, that are considered practical, and that permit consistent regulatory judgments.

### Case for a modular approach to validation.

Because of the extraordinary rate at which toxicogenomics technologies are evolving, current validation processes might need to adapt so as to accommodate the rapidly developing changes and advancements while still observing the basic tried-and-true validation principles. To meet this anticipated need, a modular approach to validation (Hartung et al. 2004) was considered, not to abridge the process but to allow for more flexibility in data collection and evaluation throughout the progressive changes that the technology will undergo. Typically, in the conventional validation procedures for an alternative test method, a sequential approach to the process is taken. The test protocol is first optimized and its transferability is determined. The resulting standardized method is then evaluated for within-lab and between-lab reproducibility and for its accuracy. Thus, an optimized, standardized protocol linked to specific test method elements and a prediction of outcome for given classes of chemicals are evaluated together for performance characteristics and applicability. Such a linear validation model, although effectively employed for other test methods, might not be optimal for dynamic test methods in which changes are rapidly introduced that improve or alter the protocol or the technology incorporated in the protocol in any substantive way. The linear validation model might result in unnecessary delays in incorporating innovations into toxicogenomics-type test methods. In contrast, with a modular approach to validation, which capitalizes on the fundamental classic concepts of validation as defined by ECVAM and ICCVAM (Balls et al. 1995; ICCVAM 1997, 2003), the different steps in the validation process are subdivided into independent modules, each of which can be assessed individually so that those components that have been completed need not undergo repeated validation. Further validation activities would instead be directed to only that part of the process flow where needed. The proposed model would accommodate validation of innovation affecting only a particular part of the sequence such that incorporation of advancements in a particular sector into testing strategies would less likely be impeded. At the same time, a modular approach to validation could efficiently handle information/data gaps that could be filled over time without derailing the validation stages already achieved. The modular approach, complemented with the use of performance standards (see “Validation as a Result of Procedural Changes” above), is expected to facilitate and help expedite the validation of the toxicogenomics technology and test methods that are based on toxicogenomics.

The modular approach follows the fundamental classic concepts of validation as defined by ECVAM and ICCVAM. Validation is defined as the process by which the relevance and reliability of a test method for a specific purpose are determined (Balls et al. 1995; ICCVAM 1997, 2003). Adequate validation involves development of a standardized test method protocol and assessment of the protocol’s within- and between-laboratory variability, predictive capacity/accuracy, usefulness and limitations, and adherence to performance standards.

### Standards for comparison.

As technologic advancements are made and new, modified, or revised toxicogenomics-type test methods are put forward for consideration, it will be necessary to have a means by which the performance of proposed methodologies can be compared with that of existing (traditional and nontraditional) methods, especially those that employ animals. The lack of an approach rooted firmly in high-quality science could jeopardize attempts to seek or gain regulatory acceptance of toxicogenomics-based test methods and strategies. Evaluations of test method performance might be based on comparisons made between particular parameters, as dictated by the specific intent for which the assay was developed. Examples include the following:

- *In vivo*–*in vivo* study comparisons to examine concordance of gene changes with such factors as onset, duration, severity, dose, age, possible temporal changes of effects, and species differences
- *In vitro*–*in vivo* study comparisons to explore gene changes associated with a critical event or end point in an *in vitro* cell-based assay and an established *in vivo* biomarker of toxicity
- *In vitro*–*in vitro* study comparisons to analyze the responses of human and animal cell systems to xenobiotics
- Technologic comparisons to evaluate the effects of proposed technical improvements (e.g., comparing gene changes using different techniques of array/platform preparation)

Accordingly, to detemine the appropriate types of validation activity and comparison in a given situation, it is important that the specific purpose of the proposed methodology and a detailed description of all relevant procedures be clearly elaborated (Balls et al. 1995; Hartung et al. 2004; ICCVAM 1997, 2003).

### *Toxicogenomics data from* in vitro *systems and data relevance*.

At the present time, toxicogenomics data derived from *in vitro* systems have been considered to have limited utility in regulatory applications. However, a great deal of interest exists for the further development of *in vitro*–based toxicogenomics methods, for an examination of their potential applicability in the regulatory arena, and for an appraisal of their potential for contributing to improvements in animal welfare. It is anticipated that technologic advancements will ultimately facilitate the use of *in vitro*–based methods as adjuncts to or surrogates for *in vivo*–based methods. Possible areas where validated *in vitro*–based toxicogenomics test methods might play a future role include *a*) preliminary assessments (prescreens), *b*) complementary testing that might assist in obtaining additional (e.g., mechanistic) information, and *c*) surrogate tests that could help in the refinement, reduction, and replacement of animals used for omics-based or traditional testing methods. One exciting aspect of toxicogenomics technology is the prospect of being able to identify species differences and/or similarities in the response to a xenobiotic. Although this is not viewed as near-term prospect, it obviously has potential applications for hazard and risk assessment purposes and could also have an impact on previous regulatory decisions when the technology becomes sufficiently advanced to permit such uses for it.

### Additional regulatory acceptance issues.

**Figure 2:**
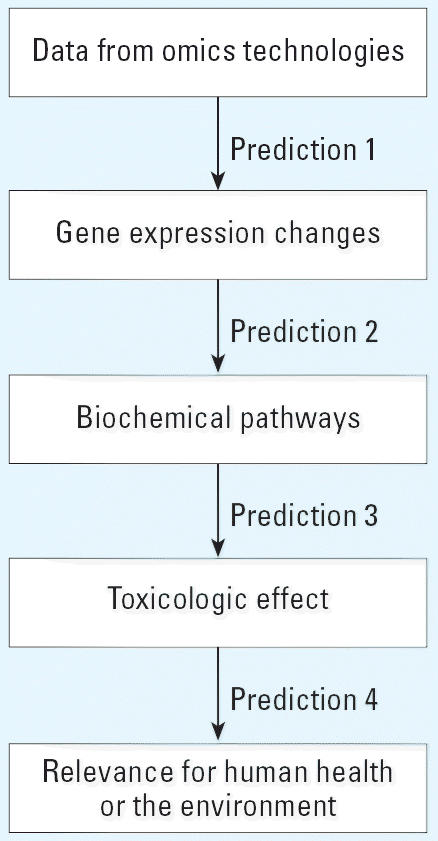
Process flow showing different independent prediction levels considered important in assessing validity of a toxicogenomics-based test method.

In considering approaches to validation, achieving regulatory acceptance of toxicogenomics-based methods or acceptance of information/data derived from such methods is an important goal. Regulators will be asked to evaluate whether data submitted using omics technologies can be used in support of a particular or broader based toxicologic, pharmacologic, or physiologic premise. For example, experiments using microarrays demonstrated increased expression of a cluster of related genes that was associated with enhanced activity and production of a microsomal enzyme important in the metabolic activation of a chemical to a toxic entity, which in turn was associated with a histopathologic biomarker lesion in the liver with a known human cancer correlate. Each of the events in this example can be thought of as a sequence of separate critical steps or information levels (Figure 2) that progressively connect omics data (from microarrays) to gene expression changes (increased expression), to a biochemical pathway (liver enzyme induction leading to toxic metabolite formation), to a toxicologic effect *in vivo* (liver lesion) with human relevance (cancer). Moving between two levels involves a prediction of outcome linking both steps. At each of these prediction junctures, regulators would be looking for evidence to scientifically substantiate moving to the next step and whether the prediction linking the levels (e.g., in this example, prediction 1, 2, 3, or 4 in Figure 2) was adequately validated. Theoretically, with this type of system, validated links could be established between any two levels. Technologic advancements or new information could be independently incorporated into a given level and considered and evaluated for the specific relevant prediction juncture. In this way, each of the prediction levels can be assessed independently and the validity of the links determined.

In the future toxicogenomics-based test methods may be shown to have been adequately validated and technically suitable for certain specific purposes, but regulatory acceptability and implementation will depend partly on whether the methods validated can be used for a given regulatory agency or program, that is, they are applicable to the products that fall within their regulatory purview. Some regulatory bodies may have internal peer-review processes, specific regulatory mandates, and/or regulatory assessment procedures that also have a role in the determination of test method applicability in regulatory programs, even though a test method may have been appropriately validated.

The widespread use of omics technologies will also bring about increasing demands on the regulatory community in terms of training of regulatory personnel in areas such as potential applications; data QC, analysis, and interpretation; statistical analysis; limitations of the technology; and how the information might be incorporated into safety, hazard, and risk assessment processes. To satisfy these needs, regulatory agencies have been engaging in developing and implementing training procedures, hiring scientists with the necessary technical knowledge and experience, establishing centers of excellence and dedicated laboratories focused specifically on the various omics and related informatics areas [e.g., National Center for Toxicological Research (U.S. FDA), NCT (NIEHS), Minister of Health Labour and Welfare–National Institute of Health Sciences Project in Japan, Netherlands Genomics Initiative, and EMBL–EBI, where informatic scientists are working with experimental practictioners and the MGED Society to ensure that transcriptomic experiments can be mapped on to regulatory toxicology studies]. In addition the regulatory arena has found that maintenance of open lines of communication with appropriate external scientists facilitates cooperation and the sharing of technical aspects, skills, and practical experiences that help to broaden the collective knowledge base. Regardless, as the technology evolves further and finds wider application and acceptance, it will be necessary to address such fundamental matters as *a*) the generation, management, and interpretation of massive amounts of data; *b*) the consequent complex questions that will undoubtedly arise (e.g., what constitutes an adverse effect as identified using the technology; how does a given gene pattern correlate with a particular toxic end point or relate to onset, duration, and severity of effects, and to age, dose, and species?); and *c*) the limitations to the technology. Addressing such issues efficiently will warrant an ongoing dialogue between regulators and practitioners and a willingness to share relevant experiential and theoretical knowledge. Standard submission and presentation formats compatible with electronic data submission likely would need to be developed. Programs and staff would need to learn how information from the new technologies might be incorporated in regulatory practices and decision-making processes and would also have to face possible incongruities between toxicogenomics-derived data and existing or future submissions of conventional toxicity data. A number of regulatory authorities have already begun to contemplate and make provisions for this enormous and challenging task, but others may not yet have committed the resources to do so.

The recommendations related to regulatory acceptance and use of toxicogenomics-based test methods are listed in Table 3.

## Conclusions

This workshop was organized as a result of the rapid growth and technologic advancements in the field of toxicogenomics; the promise it offers for numerous scientific arenas, especially human health and the environment; and the interest demonstrated by regulatory agencies as well as by the industrial sector. Consequently, it has become apparent that a considerable effort needs to be invested in the appropriate validation of both the technology alone and those test methods that incorporate the technology. The workshop provided a platform for technical experts in the field to become cognizant of the validation principles and regulatory issues to be encountered and for regulators and principal validation bodies to gain a better sense of those technologic aspects that would lend themselves to standardization, harmonization, and validation. Thus, this workshop was an important initiative that fostered an exchange of information fundamental to the ultimate adoption of toxicogenomics-based test methods for regulatory decision-making purposes. It is envisioned that the conclusions and recommendations that resulted will be a basis for future validation considerations for test method applications of toxicogenomics technologies in the regulatory arena and evaluating their potential utility for hazard/safety/risk assessments.

Several aspects of the validation of toxicogenomics that were identified as needing further exploration to help facilitate regulatory acceptance of future toxicogenomics-based test methods are as follows:

- Conduct toxicogenomics-based tests and the associated conventional toxicologic tests in parallel to *a*) generate comparative data supportive of the use of the former in place of the latter or *b*) provide relevant mechanistic data to help define the biological relevance of such responses within a toxicologic context
- Determine and understand the range of biologic and technical variability between experiments and between laboratories and ways to bring about greater reproducibility
- In the short term, favor defined biomarkers that are independent from technology platforms, and therefore are easier to validate; in the longer term, focus on pathway analysis (i.e., system biology approach) rather than just on individual genes
- Harmonize reference materials, QC measures, and data standards and develop compatible databases and informatics platforms that are key components of any validation strategy for a toxicologic method; this can only be achieved by promoting partnerships and collaborations among ongoing initiatives in toxicogenomics, standardization, and validation
- Determine performance standards for toxicogenomics-based test methods that will serve as the yardsticks for comparable test methods that are based on similar operational properties
- Define further the modular validation scheme that would allow keeping up with methodologic improvements and innovations without having to repeat the entire validation process but would, however, integrate ECVAM and ICCVAM principles of validation and acceptance.

## Supplementary Material

Supplemental Figures and Tables

## Figures and Tables

**Table 1:** Recommendations: focus on biological systems.

Encourage increased use of toxicogenomics-based approaches to define the mechanistic context of toxic responses to exogenous compounds Promote greater understanding of the relationships between gene expression responses and altered phenotype, considering the biological pathways affected, dose response, and the point of departure from adaptive to toxic response Favor the identification of biomarkers that are independent of technology platform but acknowledge the potential strengths of pathway analysis Characterize the range and extent of biological variability of responses for the test systems (e.g., diurnal effects, animal care and use, age-related context) Encourage the immediate use of toxicogenomics-based approaches in conjunction with conventional toxicity testing approaches Explore the extent to which toxicogenomics can address cross-species responses and specific disease states Promote the conduct of parallel and comparative *in vivo* and *in vitro* studies to identify *in vitro* systems that can serve as surrogates for *in vivo* systems Characterize predictive toxicology models with respect to parameters such as dose, time, study design, relevance; characterize the system to fulfill validation criteria Promote the identification of gene and protein biomarkers as early (prognostic) markers as a refinement to existing toxicity testing methods Establish a compendium of toxicant information based on gene expression responses for model compounds across multiple species, end points, and test systems Foster the development of effective partnerships between academic, government, and industry groups to promote collaborative efforts to validate toxicogenomics-based test methods and generate sufficient high-quality data to support regulatory decision making

**Table 2:** Recommendations: focus on technology.

Validation and QA/QC should be mandatory during the manufacturing of the arrays The array should undergo sequence verification and sequences should be available in the public domain MIAME guidelines should be adhered to Initially, develop “best practices” for toxicogenomics, including the interpretation of data and how to manage uncertainties and limitations Subsequently develop guidance for and adherence to GLPs for toxicogenomics experiments Common reference standards should be considered A workshop should be convened to address the development of standards for RNA sample preparation (and other biologic aspects of microarray analyses) Develop a “common” RNA standard including developing consensus about sources and maintenance of baseline data for regulatory and research purposes Studies should be MIAME-Tox compliant Performance standards should be developed and implemented to evaluate reliability and accuracy of test methods incorporating procedural modifications An ongoing dialogue should be maintained between scientists in the various relevant disciplines, including bioinformaticians, through meetings, published papers, and advisory/discussion panels (e.g., ILSI-HESI committee, NCT consortium, OECD panel) Ensure that validation efforts and QA/QC criteria are not restrictive to the technology or its advancement Explore whether toxicogenomics measurements can define toxicologic effects quantitatively Develop prediction models (e.g., algorithms) for toxicogenomics-based test methods Develop a data infrastructure for capturing, storing, and reporting toxicogenomics data Ensure continuation of financial support for long-term public database maintenance

**Table 3:** Recommendations: focus on regulatory acceptance of toxicogenomics-based methods.

Build on and/or learn from previous and ongoing efforts in toxicogenomics, standardization, validation, and harmonization efforts where possible (e.g., MIAME, ICCVAM, ECVAM, NCT, EMBL–EBI, ILSI–HESI, U.S. FDA, U.S. EPA, OECD) Fund pilot programs to test possible validation strategies and processes Identify training needs and assist in developing training vehicles and ways of presenting the state-of-the- science to regulators and the regulated community (including electronic means) Maintain transparency of validation processes Explore additions, amendments, and revisions to ICCVAM and ECVAM validation guidance that would accommodate new and rapidly changing technologies Implement the modular approach to validation to accommodate existing knowledge and future technical developments Establish performance standards for toxicogenomics-based test methods and have them accommodate rapid technologic advancements and procedural modifications Explore, develop, and support sector-spanning worldwide harmonization entities Create confidence among regulators by involving them early on in discussions and various scientific forums that would facilitate application of the technology for regulatory purposes Encourage industry and other parties to share data, in part, to support validation comparisons Promote high-quality science in supporting the use and development of the technology for regulatory purposes to further protection of human health and the environment Consider opportunities for synergy between QSAR, pharmacokinetic, and pharmacodynamic modeling, and other *in silico* efforts and the toxicogenomics communities
