# Supplementary material for: Meeting Report: Validation of Toxicogenomics-Based Test Systems: ECVAM–ICCVAM/NICEATM Considerations for Regulatory Use
Source: Environ Health Perspect. 2005 Aug 17;114(3):420–9. doi: 10.1289/ehp.8247 (PMC1392237; doi:10.1289/ehp.8247)
Supplement: Supplemental Figures and Tables [file ehp0114-000420s1.pdf]

Validation of Toxicogenomics-Based Test Systems

Supplemental information

Section 1 on Identification of Biomarkers

Ideally, simple biomarkers will be identified concurrently with gene expression signatures for model compounds and endpoints. Expression signatures can be correlated with biomarkers to address complex biological mechanisms. This approach is feasible provided there is sufficient knowledge of the biological pathways and mechanisms underlying the toxic response. The task is more difficult when information about mechanism is lacking. In these instances, conventional structure-activity predictions can be used in conjunction with conventional toxicity testing to infer biological mechanisms. Recent efforts by the ILSI-HESI Consortium on the Application of Genomics to Mechanism Based Risk Assessment (http://hesi.ilsi.org), and the NIEHS Toxicogenomics Research Consortium (TRC) (http://www.niehs.nih.gov/dert/trc.htm) indicate that correlation of individual gene expression measurements is variable across microarray platforms and research centers. Despite individual gene expression differences, the ILSI-HESI consortium demonstrated that a core set of biological pathways were activated in response to a specific hepatotoxin, and pathway elucidation was independent of platform (Ulrich et al. 2004).

More complete understanding of the cellular pathways and processes that contribute to a toxicological phenotype will require that individual gene expression changes be placed in

the context of the underlying biological pathways. Identification of gene and protein expression changes in relation to specific biological pathways is clearly desirable, but also limited by existing knowledge about biological processes and pathways. Available mapping tools are often limited by incomplete or inaccurate annotation of pathway components and linkages. Visual inspection of gene expression changes by gene category is not sufficient for complex multi-component pathways and networks where a significant number of gene changes will be observed due to chance (Kelley et al. 2003). Sophisticated statistical methods and improved mapping tools are needed to identify relevant biological pathways using gene expression measurements. Ultimately, knowledge of both transcriptional and translational control will be crucial to understanding the relationship between gene expression and the subsequent expression of associated proteins.

Ulrich RG, Rockett JC, Gibson GG, Pettit SD. 2004. Overview of an interlaboratory collaboration on evaluating the effects of model hepatotoxicants on hepatic gene expression. Env Health Perspectives 112(4):423-427.

Kelley BP, Karp SR, Sittler T, Root DE, Stockwell BR, Ideker T. 2003. Conserved pathways within bacteria and yeast as revealed by global protein network alignment. Proc Natl Acad Sci USA 100(20):11394-11399.

Section 2 on Data Management

Similarly, MIAME has been developed to fulfill the specific requirements of other domains of application. The MIAME/Env checklist has been developed by the UK Natural Environment Research Council (NERC) for the environmental genomics community (www.nerc.ac.uk/funding/thematics/envgen) and a MIAME/Nut checklist is being developed by the Nutrigenomics Organization (NUGO) (www.nugo.org) to fulfill the specific requirements of their array-based applications (Garosi et al. 2005). Although discipline-specific initiatives are important, ultimately this fragmentation will be detrimental. Firstly, they will create unnecessary duplications. The checklists' names categorize them strictly in a specific domain, however, the boundary across these disciplines is not so defined and some of the requirements are definitely shared. Secondly, as other –omics technologies will be used in combination with microarray these checklists will be soon insufficient to serve the scope. To maximize the synergy and optimize harmonization, a new working group has been formed within MGED recently, acting as a ‘single point of focus’ for Toxicogenomics, Environmental Genomics and Nutrigenomics communities, where efforts are already underway to promote standardization and develop databases to facilitate data exchange. The MGED Reporting Structure for Biological Investigations Working Groups (RSBI WGs) (http://www.mged.org/Workgroups/rsbi/rsbi.html) aim to maintain collaboration between technology-driven standardization efforts and activities that relate to biological investigations in specific domains of application. There are several organizations and committees that are tackling data standardization issues in a wider context of using emerging highly parallel -omics approaches. These include the Pharmacogenomics

Standards Group, a joint project of the Clinical Data Interchange Standards (CDISC), Health Level Seven (HL7) [www.hl7.org] and Interoperable Informatics Infrastructure Consortium (I3C) [www.i3c.org], including industry, software developers and governmental representatives, aiming to define the requirements for pharmacogenomics submission to FDA and clarify data formats, standards. Lastly the MGED RSBI WGs aim to develop a reporting structure for describing information intensive investigations RSBI Tiered Checklist (RSBI TC) is under way. RSBI TC will be a modular context dependent structure allowing to described the same concept unambiguously within and across the different communities.

Garosi P, De Filippo C, van Erk M, Rocca-Serra P, Sansone SA, Elliott R. 2005. Defining best practice for microarray analyses in nutrigenomic studies. Br J Nutr 93(4):425-432.
